# Setting individualised goals for people living with dementia and their family carers: A systematic review of goal-setting outcome measures and their psychometric properties

**DOI:** 10.1177/14713012231222309

**Published:** 2023-12-17

**Authors:** Jessica Budgett, Andrew Sommerlad, Nuriye Kupeli, Sedigheh Zabihi, Anna Olsen, Claudia Cooper

**Affiliations:** Division of Psychiatry, University College London, and Centre for Psychiatry 4919 and Mental Health, Wolfsen Institute of Population Health, Queen Mary University of London UK; Division of Psychiatry, 4919 University College London and Camden and Islington NHS Foundation Trust, UK; Division of Psychiatry, Marie Curie Palliative Care Research Department, 4919 University College London, UK; Division of Psychiatry, 4919 University College London, UK; Centre for Psychiatry and Mental Health, Wolfson Institute of Population Health, 4617 Queen Mary University of London, UK

**Keywords:** dementia, goal setting, goal attainment scaling, goal attainment, outcome measures, psychometric properties, family carers, systematic review

## Abstract

**Background:**

Individualised goal-setting outcome measures can be a useful way of reflecting people living with dementia and family carers’ differing priorities regarding quality-of-life domains in the highly heterogeneous symptomatology of the disease. Evaluating goal-setting measures is challenging, and there is limited evidence for their psychometric properties.

**Aim:**

(1) To describe what goal-setting outcomes have been used in this population; (2) To evaluate their validity, reliability, and feasibility in RCTs.

**Method:**

We systematically reviewed studies that utilised goal-setting outcome measures for people living dementia or their family carers. We adapted a risk of bias and quality rating system based on the COSMIN guidelines to evaluate the measurement properties of outcomes when used within RCTs.

**Results:**

Thirty studies meeting inclusion criteria used four different goal-setting outcome measures: Goal Attainment Scaling (GAS), Bangor Goal Setting Interview (BGSI), Canadian Occupational Performance Measure (COPM) and Individually Prioritized Problems Assessment (IPPA); other papers have reported study-specific goal-setting attainment systems. Only GAS has been used as an outcome over periods greater than 9 months (up to a year). Within RCTs there was moderate quality evidence for sufficient content validity and construct validity for GAS, COPM and the BGSI. Reliability was only assessed in one RCT (using BGSI); in which two raters reviewed interview transcripts to rate goals with excellent inter-rater reliability. Feasibility was reported as good across the measures with a low level of missing data.

**Conclusion:**

We found moderate quality evidence for good content and construct validity and feasibility of GAS, BGSI and COPM. While more evidence of reliability of these measures is needed, we recommend that future trials consider using individualised goal setting measures, to report the effect of interventions on outcomes that are most meaningful to people living with dementia and their families.

## Introduction

Dementia is characterised by highly heterogenous symptoms including cognitive impairments and other neuropsychiatric symptoms which impair daily functioning (WHO, 2022). Quality of life is consistently cited by older adults as more important than disease specific outcomes (Tochel et al., 2019) and is included as an outcome in many dementia trials. Because dementia symptoms and domains of quality of life are varied and of differing relevance to people living with dementia and their relatives, there is a focus on patient-reported relevant outcomes measures (PROMs) (Cooper et al., 2012).

The main alternative to standardised scaled outcome measures is to use highly individualised goal setting or goal attainment scaling systems. In this paper we define goal-setting outcome measures as those using a system to set individualised goals (brief statements about a behaviour that the user would like to carry out or achieve) with people living with dementia and/or their family carers, against which attainment can be rated. Most goal-setting measures aim to capture individualised and clinically meaningful outcomes (Shabbir & Sanders, 2014) making them particularly suited for assessing interventions for diseases with heterogenous symptoms and stages such as dementia.

A 2008 systematic review examining the utility of Goal Attainment Scaling (GAS) for people living with dementia reported mixed findings regarding responsiveness, reliability, validity and feasibility (Bouwens et al., 2008). It identified a small number of studies that used GAS, and 9/10 reviewed studies were conducted by the same research group (Bouwens et al., 2008). They concluded that the evidence was not yet strong enough to state that GAS was a suitable for this population but affirmed its potential value of being uniquely able to reflect the multidimensionality of dementia. Dementia trials are now developing a wide range of interventions (including drugs, rehabilitation, psychosocial, environmental, preventative, or different approaches to delivering care) and all are striving to be bolder in their vision for person-centred approaches to dementia care (Kim & Park, 2017). To our knowledge, there has been no more recent, nor broader review of all goal-setting measures for people living with dementia or their family carers.

Goal setting outcome measures differ from conventional PROMs as they lack fixed items. The construct being measured is commonly described as ‘the change’ or extent to which the goal is achieved because of an intervention on an aspect of the user’s life. The number of goals, goal content and attainment levels vary between studies and participants, so validity and reliability are complex to measure (Gaasterland et al., 2019). In this review, we follow the *COnsensus-based Standards for the selection of health Measurement Instruments* (COSMIN) guidelines (Mokkink et al., 2020), a comprehensive checklist designed to assess the psychometric properties and methodological quality of outcome measures. Many of the COSMIN criteria cannot be evaluated for goal-setting outcome measures, including criterion validity and internal consistency. A review of GAS within drug trials used an adapted version of COSMIN and found that the included trials reported on inter-rater reliability, content validity, construct validity and responsiveness of goal setting measures (Gaasterland et al., 2016). Gaasterland et al. (2019) provide guidelines of how to evaluate content validity, construct validity and inter-rater, intra-rater and inter-trial reliability of goal-setting outcomes, which we have used in this review (Table 1).

**Table 1.** COSMIN (Mokkink et al., 2010) and goal outcome-adapted COSMIN definitions of measurement properties, and their quality criteria (Gaasterland et al., 2016, 2019).

Measurement property	COSMIN definition	Adapted definition for goal-setting outcome measure	Quality criteria (+ is good/sufficient quality, +/− is indeterminate quality and – is insufficient quality)
Content validity	Degree to which instrument content adequately reflects of construct measured	Degree to which goals are clearly identified, defined and capture outcomes relevant to the intervention AND scoring is consistent, centred around an appropriate baseline value and proportionally ordered	+ A clear description of the measurement aim, target population, concepts measured, and item selection procedures involving the target population, and experts consulted
			+/− clear description of these aspects lacking
			− Not clear
Construct validity (individual level)	Degree to which instrument scores are consistent with hypotheses, and measure the target construct	The underlying construct(s) is the attainment of goals appropriate to the intended intervention effect. Evaluated through comparison of change scores on individual goals with measurements that capture similar constructs	+ Specific hypotheses were formulated and correlation coefficients between goal outcome and comparison outcomes is high (>.69)
			+/− doubtful design or method AND/OR modest correlations
			− Less than 75% of hypotheses were confirmed AND/OR low correlations (<.5)
Construct validity (trial level)		Construct validity can be evaluated through mean scores of two randomised groups, testing hypotheses that these will differ in favour of the group receiving the effective intervention	+ Specific hypotheses were formulated and at least 75% of the results are in accordance with these hypotheses
			+/− doubtful design or method (e.g., no hypotheses) unclear
			− Less than 75% of hypotheses were confirmed
Intra-rater reliability	Extent to which scores for participants who have not changed are comparable when repeated by the same raters on different occasions	Goal attainment is assessed consistently when performed by the same rater for the same participant in the same condition repeatedly (possibly hypothetically)	+ ICC or weighted Kappa ≥.7 AND/OR repeated assessment of video recording of the assessment of goal attainment by the same rater
			+/− unclear design or method
			− ICC or weighted Kappa ≤0.7
Inter-rater reliability	Extent to which scores for participants who have not changed are comparable when repeated by different raters on the same occasion	Goal attainment is assessed consistently when performed by different raters for the same participant in the same condition	+ ICC or weighted Kappa ≥.7 AND/OR assessment of the video recording of the goal attainment scoring by one or more independent raters
			+/− unclear design or method
			− ICC or weighted Kappa ≤0.7
Inter trial reliability		Goal attainment scaling leads to consistent results when implemented in repeated implementations of the same trial	+ Replication between trials or within one trial (split-half design) is demonstrated shows good comparison of mean difference between groups

Abbreviations: ICC = intraclass correlation coefficient.

In this paper we aim to evaluate evidence regarding the utility of goal-setting outcome measures in people living with dementia and their family carers. Our aims are to (a) describe what goal-setting measures have been used with people living with dementia and their family carers; and (b) evaluate the validity (content and construct), reliability (inter-rater reliability and responsiveness) and feasibility of measures that have been used in RCTs.

## Methods

We registered our protocol on the Prospective Register of Systematic Reviews (PROSPERO - CRD42021245401) and used PRISMA guidelines (Moher et al., 2015) to conduct and report this review.

### Search strategy

In February 2022 we searched CINAHL, Embase, PsychInfo and Medline for studies that used individualised goal focused outcome measures for people with dementia and/or their family carers. The databases were examined using a combination of keywords within three blocks: (1) Dementia, (2) Goals, and (3) Outcome Measures, with synonyms and relevant MeSH headings tailored to each database (full search details in Appendix 1).

### Inclusion and exclusion criteria

Titles and abstracts were screened by the first author (J.B.) and 10% were independently reviewed by second reviewer (C.C.) to identify articles where one or more individualised goals were set for people living with dementia and/or their family carers and used as outcome measures for any type of intervention. We included studies where ≥75% of the sample had a diagnosis of dementia. One researcher (J.B.) then reviewed the full texts to select the final eligible articles, discussing uncertainties with the wider research team. Studies were included where;

• Goals were set and rated by either a family carer, person living with dementia, clinician, researcher, or a combination of these.
• At least one psychometric property (validity or reliability) was assessed or the feasibility or interpretability of the goal-setting outcomes was reported.

We excluded case studies, dissertation abstracts, protocols, and reviews.

### Data extraction

We used Covidence, a web-based collaboration software platform that streamlines the production of systematic reviews, for data management. Duplicate articles were removed. See Table 2 for details of data extracted.

**Table 2.** Outline of the study populations, Interventions, goal setting outcome measure methods, follow up periods and reported findings and measurement properties in the included studies.

Study/Design	Study population	Intervention	Details of goal setting	Follow-up	Measurement properties and findings
Goal attainment scaling (GAS)					
(Rockwood et al., 1996) RCT	15 Canadian patients with Alzheimer’s disease from geriatric outpatient clinic	Drug; Linopirdine	Clinicians facilitated setting/scoring goals with family carers (and patients if possible). 5-Point scale (−2 to +2), −2 or −1 was baseline. 3.7 (2–6) goals set per patient	6 months^a^	No significant difference in GAS between allocated groups (*p* = .54). GAS had largest effect size (.61) and relative efficacy (.47) compared to other measures. Mixed results for responsiveness and construct validity, unclear content validity. Good acceptability. GAS setting took 2 hours/3 visits
(Hartman et al., 1997) prospective descriptive study	10 Canadian men with dementia from special care units	Program of occupational therapy and therapeutic recreation	Occupational therapists and therapeutic recreation specialists set/scored goals. Family carers provided information related to premorbid levels of function and interests. 5-Point scale (−2 to +2), 0 was ‘expected level of outcome’. No weighting. ≤2 goals set per person with dementia	Following each goal related activity and at 3 months^a^	GAS was feasible (goal setting took average of 20 minutes) and responsive to change (effect size = 2.34). GAS was reported to have focused treatment planning
(Gordon et al., 1999) prospective descriptive study	53 Canadian nursing home residents (41 with dementia)	Specialised geriatric medicine consultation	Two geriatricians and nurse collaborated to set/score goals. 5-Point scale (−2 to +2), −2 or −1 was baseline (−2 if deterioration considered not possible). 89 goals set (1.7, per patient)	Residents monitored (mean = 44 days), until goal attained or determined unachievable^a^	GAS was feasible; and more responsive (higher effect size (1.29) and highest relative efficiency (53.7)) than BI and other measures. Low correlation with other measures (−.22 to .17) indicating low construct validity but may capture different information
(Rockwood et al., 2002) prospective study	108 Canadian patients with Alzheimer’s disease from the community	Drug; donepezil	Health professional facilitated/scored goals with patients and family carers. Physicians set their own goals for the patients. 5-Point scale (−2 to +2), 0 was baseline. Patient and family carer goals set were weighted on visual analogue scale in order of relative importance. Physician goals were not weighted. 855 goals set (9 per patient)	12, 24, 36 and 52 weeks^a^	Good construct validity inferred by correlations with standard measures. Patient/family carers set more goals (mean = 9) than physicians (3) and different goals: More relating to leisure, social interaction, function, and behaviour; fewer cognition.
(Rockwood et al., 2006, 2007) RCT	130 Canadian patients with Alzheimer’s disease from the community	Drug; galantamine	Two independent goal-setting assessments; one by physicians after interviewing patients and family carers (377 goals set), the other by patients and family carers in interview by experienced, independent health professional (429 goals set). 5-Point scale (−2 to +2), 0 was baseline	Every 8 weeks for 8 months by both raters^a^	Physician-rated, but not patient/carer-rated goals, improved with treatment. GAS content validity shown by blinded raters coding video recorded interviews to assign goal domains
(Leroi et al., 2014) RCT	25 UK patients with Parkinson’s disease dementia from the community	Drug; memantine	Psychiatrists and RAs facilitated setting/scored goals. Goals set with family carers and patients (if possible). Problems were identified within five domains: Behavioural, leisure, functional, motor, and cognitive states. 5-Point scale (−2 to +2), −2 or −1 was baseline level (−2 if deterioration considered not possible). Goals not weighted. 30 goals set (2.8 goals set per patient)	16 weeks^a^	GAS responsive to change -improvement shown by GAS in the treatment group was not replicated on other psychometric tests
(Ciro et al., 2014) quasi-experimental	16 USA community patients with mild/moderate dementia	Task-oriented motor practice (STOMP) intervention	OTs set/scored goals; they identified key tasks with family carers using the COPM and rated each from 1 (unable) to 10 (fully able to perform). Goal areas identified were then formatted using GAS 5-point scale (−2 to +2), 0 was ‘expected level of outcome’. Time to achieve goal recorded	1 week and 3 months	GAS-T scores improved significantly from pre -to post- intervention and worked equally as well in the home or in the clinic
(Schinköthe et al., 2015; Wilz et al., 2011); RCT (GAS in intervention)	126 German family carers from the community	Cognitive behavioural therapy (CBT)-based telephone intervention for family carers	Psychotherapists facilitated setting/scored goals. Goals set with family carers during intervention; 5-point scale used (−1 to +3); 0 was the baseline level. Goals ranked for importance	Therapist and family carers at 3 months (final session)	30.1% achieved complete goal attainment, 39.8% partial goal attainment, and 24.1% no change. Good feasibility, most goals set in the first or second session. The therapist’s CBT competency but not manual adherence predicted higher GAS
(Chew et al., 2015) non-randomised study	44 singaporean people with dementia and family carers from outpatient geriatric clinics	Multidisciplinary rehabilitation programme (MINDVital)	Nurse coordinator facilitated setting/scored goals with people with dementia and family carers. 5-Point scale (−2 to +2), 0 was ‘expected level of outcome’. No weighting. ≤2 goals set per person with dementia	8 weeks^a^	62% of participants met or exceeded goals. GAS significantly correlated with improved caregiver burden (ZBI) and behaviour severity (NPI-Q)
(Boots et al., 2016) non-randomised pilot study	28 Dutch family carers from clinics, community, and care homes	Self-management program for self-efficacy and goal attainment	Experienced professional coach facilitated setting/scored goals with family carers. 5-Point scale (−2 to +2), 0 was ‘goal attained’. Goals weighted. 13 goals/8 family carers (1.6 per family carer)	8 weeks^a^	GAS *T* score significantly improved after intervention. 8 goals attained and 3 goals were unattained
(Boots et al., 2017) RCT (GAS only in intervention group)	48 Dutch early dementia family carers from memory clinics and family carer support services	Self-management program, “partner in Balance” (PiB)	Experienced professional coach facilitated setting/scored goals with family carers. 5-Point scale (−2 to +2), 0 was ‘goal attained’. Goals weighted. 93 goals/42 family carers (2.2 per carer)	3, 6 and 12 months^a^	Most participants (*n* = 35) achieved ≥1 goals. 5 participants were not able to set goals
(Petyaeva et al., 2018) feasibility study	3 care homes (all staff) and 19 Canadian patients with dementia	PAIN-Dem intervention (pain management training and support)	Trained researcher facilitated setting/scored goals. ≤3 goals set with care home staff and patients. Goal attainment rated as 0%, 25%, 50%, 75% or 100%	4 weeks	Goal attainment in residents significantly improved, although no significant change in pain was seen on a pain scale. Good feasibility – completion of GAS by care staff was 100%
(Jennings et al., 2018) observational	101 USA people with dementia and caregiver dyads	GAS process within dementia care management program	Dementia care managers (DCMs) facilitated/scored foals. Goals selected by dyads from a goal inventory (Jennings et al., 2018). DCMs formed GAS goals. 5-Point scale (−2 to +2); −2 or −1 was baseline level (−2 if deterioration considered not possible). DCMs rated how difficult they thought the goal would be to achieve on 4 response scale (not at all to extremely difficult). 112 goals set. Family carers had option to change goals/revise scaling at 6 months	6- And 12-month	74% of participants attained goals. Almost all goals selected were rated as extremely (83%) or very important (13%) by the dyad, and as a little (43%) or moderately difficult (32%) by DCMs. Only 11 participants (13%) revised the scaling of their goal or chose a new goal at 6 months. GAS took 15–20 mins on average in clinic
(Wilz et al., 2018) secondary analysis of RCT	139 German family carers from the community	CBT-based telephone intervention for family carers	Psychotherapists facilitated setting/scored goals with family carers during intervention. 5-Point scale used (−1 to +3); 0 was baseline. Goals were ranked in order of importance	End of therapy session (3 months)	20.9% exceeded, 56.4% completely attained, and 21.8% partially attained at least one of their personal goals. High agreement between self- and therapist ratings
(Berwig et al., 2020) feasibility study	5 German patients with dementia and family carers from community	Marte Meo® (MM) counselling	MM researcher facilitated setting/scoring goals with the family carers. 5-Point scale (−2 to +2), 0 was ‘expected success’. 5 goals set (1 per carer)	8 weeks	3 family carers reached their goals more than expected and 2 carers reached their goals as expected
(Harris et al., 2020) feasibility trial	11 UK people with dementia and carers from the community	A tablet-based prompter tool	OT researcher facilitated setting/scoring goals with people with dementia and carers (max 2 goals for person with dementia and 1 for carer). Modified 3-point scale used: 0 (baseline/no change); +1 (partially achieved); +2 (completely achieved). Combining points from the dyad’s 3 goals provided a score from 0 to 6.22 goals set.	4 weeks post intervention	1/11 dyads did not rate themselves as having either fully or partially met at least one of the goals that they had set. For people with dementia, 14 goals fully met, 1 partially met and 7 not met. For carers, 8 goals were fully met, 2 partially met, 1 not met.
(Watchman et al., 2021) participatory action study	16 UK people with dementia and intellectual disability (ID) from social care services	Psychosocial interventions based on needs	Social care staff facilitated setting/scoring goals with participants. Goals set by participants with capacity and collaboratively if lacked capacity. Scale not outlined	6 months^a^	In participants with capacity, 32% of goals were either met and 43% exceeded expectations. In participants without capacity, 35% of goals were met and 37% exceeded expectations
(Chester et al., 2021) RCT (GAS only for intervention group)	117 UK people with dementia (early stage) and their carers from the community	DESCANT intervention (provision of cognitive aids via technology)	Dementia support practitioners (DSPs) facilitated setting/scored goals with people with dementia and family carers. 5-Point scale used, 0 was baseline. 293 goals set.	1 and 4 weeks^a^	Mean GAS score of 1.4 (SD 0.6 and possible range between −2 and 2) strongly suggests that participants improved against their chosen goals. Goal attainment scored for 95% of dyads. Inter-rater reliability checked and GAS assessed as feasible for use with psychosocial interventions
(Rapaport et al., 2021) feasibility trial	16 UK patients with dementia and their carers from memory clinics	NIDUS-family intervention (manualised and individualised psychosocial support)	Non-clinically trained researchers facilitated setting/scored goals with family carers and patients (where possible). 5-Point scale used (−2 to +2), 0 was baseline level. Goals selected within domains from a goal inventory. Goals reviewed by study team before randomisation. 57 goals set (3.9 per dyad)	6 months^a^	Mean GAS scores (59.0 (95% confidence interval 49.5–68.5)) exceeded baseline expectations (50 = met baseline expectations). Goal setting was part of the success of the intervention, yet inflexibility in changing goals during the intervention could be limiting
Canadian occupational performance measure (COPM)					
(Clare et al., 2010, 2011) RCT and subgroup analysis	69 UK patients with Alzheimer’s disease, mixed or vascular dementia from memory clinics	Cognitive rehabilitation (CR)	A trained and blinded research assistant facilitated goal setting/scored up to 5 goals with the person with dementia in areas related to self-care, leisure, and productivity. 208 goals set (mean 3.5 per dyad). Level of performance and level of satisfaction of goals were rated on scale of 1; unable to perform/not satisfied, to 10; fully able to perform/extremely satisfied	Post intervention at 8 weeks	Performance and satisfaction were strongly correlated (r = .712, *p* < .001); and improved more in CR group. Carer involvement led to improved performance. 12 goals (46%) were rated as fully achieved, 13 (50%) as partially achieved and 1 (4%) as not achieved
(Regan et al., 2017) RCT	55 Australian people with dementia and their carers from memory clinics	Cognitive rehabilitation intervention (MAXCOG)	A trained and blinded research assistant facilitated goal setting/scoring with people with dementia to identify up to 5 goals. 143 goals set (mean 3.7 per dyad). Level of performance and level of satisfaction of goals were rated.10-point scale used (as above)	Baseline and 4 weeks	Good feasibility, participants able to identify ≤1 goal. Intervention group significantly higher performance and satisfaction with primary goals post-intervention than the control group
Bangor goal-setting interview (BGSI)					
(Hindle et al., 2018; Watermeyer et al., 2016) single blind pilot/RCT	29 UK patients with Parkinson’s disease dementia or dementia with Lewy Bodiesfrom movement disorder and memory clinics	Cognitive rehabilitation (CR), relaxation therapy (RT) or treatment as usual (TAU)	Researchers facilitated setting/scoring up to 3 SMART goals with the PLWD and their FC (if available) via BGSI interview. PLWD and FCs rated their goal attainment and satisfaction with goal attainment at baseline, on a scale of 1; unable to perform/not satisfied, to 10; fully able to perform/extremely satisfied	2 months and 6 months	Excellent interrater reliability (Krippendorff’s alpha is .95), agreement 95.7%. PLWDs’ mean ratings for goal performance was significantly higher than FCs’ mean ratings. Goal setting deemed feasible for patients with PDD or DLB. Significant improvement in goal attainment in the CR group compared to the RT and TAU groups
(Clare et al., 2019) RCT	475 UK patients with dementia and family carers from memory clinics	Cognitive rehabilitation intervention	Research assistants facilitated setting/scoring goals. BGSI interview used to ask patients and family carers how cognitive difficulties affect (1) everyday tasks, activities, and routines, (2) engaging in pleasurable and meaningful activities and (3) social contacts and relationships. Patients and family carers made parallel ratings on a scale from 1; unable to perform/not satisfied, to 10; fully able to perform/extremely satisfied. 1358 goals set, 87.6% set 3 goals	3 and 9 months	Participants in the CR group showed goal attainment at 3 months which was further maintained at 9 months. Difference between the control and rehabilitation groups showed large effect size (Cohen’s d .81). Secondary outcomes showed no statistically significant differences between the groups, with small or no effect sizes. Readiness to change in relation to the set goal was significantly associated with goal attainment
(Kelly et al., 2019) descriptive pre-post study	3 Irish patients with Alzheimer’s disease	Cognitive rehabilitation intervention	Researchers facilitated setting 3–4 goals with the patients and family carers using the BGSI interview. The patients and carers rated their performance and satisfaction with these goals on the 1–10 scale. 12 goals set.	11 weeks (1 week after intervention) and 17 weeks	Goal performance and satisfaction improved for all participants with only 1 out of 12 goals scoring the same at baseline and post-test
Individually prioritized problems assessment (IPPA)					
(Bemelmans et al., 2016) quasi-experimental study	91 Dutch residents with dementia from care homes	‘Paro’ (a socially assistive seal robot) use for therapeutic purposes or daily care activities	Facilitator not specified. IPPA interview conducted to identify the daily activity problems for the residents. Residents rated the problem on a 5-point scale from 1; not important at all/no difficulty at all to 5; most important/too much difficulty to perform the activity at all. The ‘difficulty scores’ are added up using the ‘importance scores’ as weighting factors and then divided by number of problems identified to provide total IPPA score for each participant (1–25)	Baseline, and every month up to 4 months	The IPPA and mood scores show a high correlation (.68). Paro showed significant effect for interventions aiming for therapeutic effects but not for interventions aiming for care support
Trial specific goal-setting measures					
(Tappen, 1994) RCT	240 USA nursing home residents (80+% with dementia)	Skill training or stimulation approach	A gerontological nurse practitioner in consultation with a physical therapist facilitated setting/scoring goals. Five goals set related to activities of daily living for each resident at baseline. 4-Point scale of 0 (decline) to +3 (great improvement). Total score was the mean of 5 goal ratings per participant	20 weeks	Skill training has the highest mean goal score followed by the stimulation group and the control group. There was a significant difference between skill training group over control group (*p* = .05)
(Bourgeois et al., 2003) quasi-experimental study	25 USA people with dementia from day care or nursing homes	Spaced retrieval (SR) and modified Cueing Hierarchy (CH) training approaches	Speech-language pathologists (SLPs) or student SLPs facilitated setting/scoring goals. Problems identified by SLPs interviewing program staff and family carers and then goals set by the SLPs. SLPs assigned goals to SR and CH procedures with specified use of external memory aids. SLPs recorded the number of sessions until goal achievement and the length of goal maintenance	1 week and 4 months	Significantly more goals were attained using SR procedures than CH. No significant differences in the number of sessions needed to attain goals in either group, but significantly more SR goals maintained at 1-week and 4 months post training
(Judge et al., 2011) quantitative descriptive study	93 US veterans with dementia and their family carers	Telephone-based care coordination intervention ‘partners in dementia care’ (PDD)	Care coordinators facilitated setting/scoring goals. Goals set with the veterans and carer dyads. Action steps/behavioural tasks were then set with the dyads to help them move towards their goals as part of a written ‘individual action plan’. 488 goals set (5.2 per dyad). Action steps rated as ‘accomplished’, ‘still in progress after 12 months’ or ‘not relevant’	Status of the action steps checked regularly (average of 24.6 contacts in 12 months)	Interrater reliability of goal setting was established at 91% agreement. Most goal action steps were assigned and completed by family carers and majority (59%) were successfully accomplished
(Kerssens et al., 2015) feasibility study	7 USA patients with dementia and their carers from the community	Personalised psychosocial interventions via touchscreen technology	A researcher facilitated setting/scored goals with family carers. During the ‘care needs’ interview where 4 symptoms were selected to be the target of the intervention and the baseline description of goal. 25 goals set. Target symptoms measured on a 4-point scale (better, stable, worse, or not applicable) and the expected level of attainment on a 5-point scale (much less than expected to somewhat more than expected)	Post intervention (minimum 3 weeks)	Post intervention, 11 (44%) of 25 goals were as expected or better than expected, whereas 8 (32%) goals were less than expected in the eyes of caregivers
(Zarit et al., 2021) non-randomised controlled study	96 UK people with dementia inpatient rehabilitation departments	iN2L technology system in rehabilitation programmes	An occupational therapist and physio therapist facilitated goal setting/scoring. Goals identified with patients through discussions based on baseline assessments (functional independence measure (FIM) and Barthel index (BI)). Ratings were made on a 10-point scale from 1; unable to do to 10; performing 100% of the task. For each goal, an expected level of functioning to be attained by the end of treatment was set. Goal attainment scored by research assistants by comparing initial and final functioning on FIM and BI items identified as goals. A dichotomous rating was used; 0 = not attained and 1 = Attained or exceeded	End of treatment (mean of 23 days)	Goal attainment was reached across both groups, although the iN2L group has significantly higher attainment than treatment as usual. Increases in engagement significantly mediated the treatment effects on goal attainment

^a^GAS score calculated using the standard formula [11].

### Assessing risk of bias and quality ratings of evidence

We evaluated methodological quality, validity, reliability and feasibility for goal outcome measures within RCTs. To do this, we adapted the COSMIN risk of bias checklist (Mokkink et al., 2018) (Appendix 2), and ‘Good Measurement Properties’ COSMIN criteria (Mokkink et al., 2010) (Table 1). We adapted Box 2 (Content validity), Box 6 (Reliability) and Box 9 and 10 (Construct validity) from the COSMIN risk of bias checklist using the standards for content and construct validity developed from Gaasterland et al. (2019, 2016)’s papers and drew on the definitions and the findings in two previous GAS reviews (Bouwens et al., 2008; Shankar et al., 2020). We distinguished between individual level and trial level construct validity (see Table 1 for definitions). Appendix 2 shows the full adapted boxes, standards, and rating guide. Each study was independently rated by two of three researchers (J.B, S.Z and A.O). For each study, we evaluated (1) risk of bias of the evaluation as ‘very good’, ‘adequate’, ‘doubtful’, or ‘inadequate’; and (2) the quality rating as sufficient, insufficient, indeterminate, and inconsistent. Any conflicting ratings were discussed as a team to reach a consensus rating. The overall study quality was recorded as the lowest rating of any standard within the box following ‘the worst score counts’ principle of COSMIN (Mokkink et al., 2018).

We extracted information related to the interpretability and feasibility of the goal setting outcomes following the COSMIN recommendations (Mokkink et al., 2020). Interpretability refers to the degree qualitative meaning can be assigned to the single scores or change in scores of the measure and included looking at completion rates and the percentage of missing data. The feasibility of the measures includes any details related to the ease of application of the measure including completion time, cost of measure use, training needed and ease of administration (Mokkink et al., 2010).

Finally, we used a modified GRADE approach (Prinsen et al., 2018; Terwee et al., 2018) to give overall ratings for the quality of the evidence (high, moderate, low, very low evidence) for content validity, construct validity and reliability of each goal setting measure.

## Results

### Search strategy results

**Figure 1.**
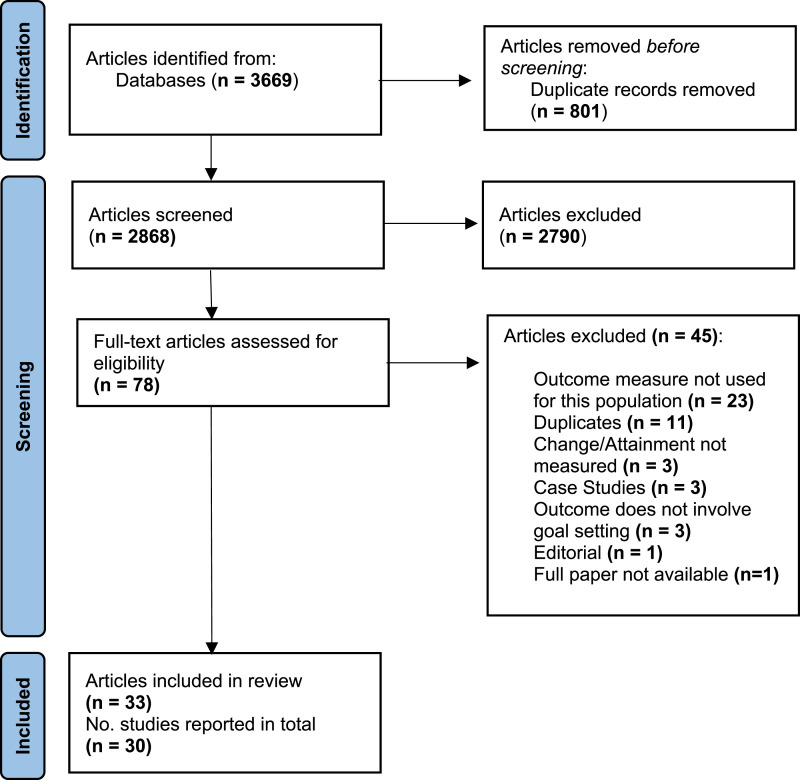
PRISMA diagram.

As outlined in Figure 1, we identified 33 articles that met the inclusion criteria, which described 30 studies.

### Overview of included studies

**Figure 2.**
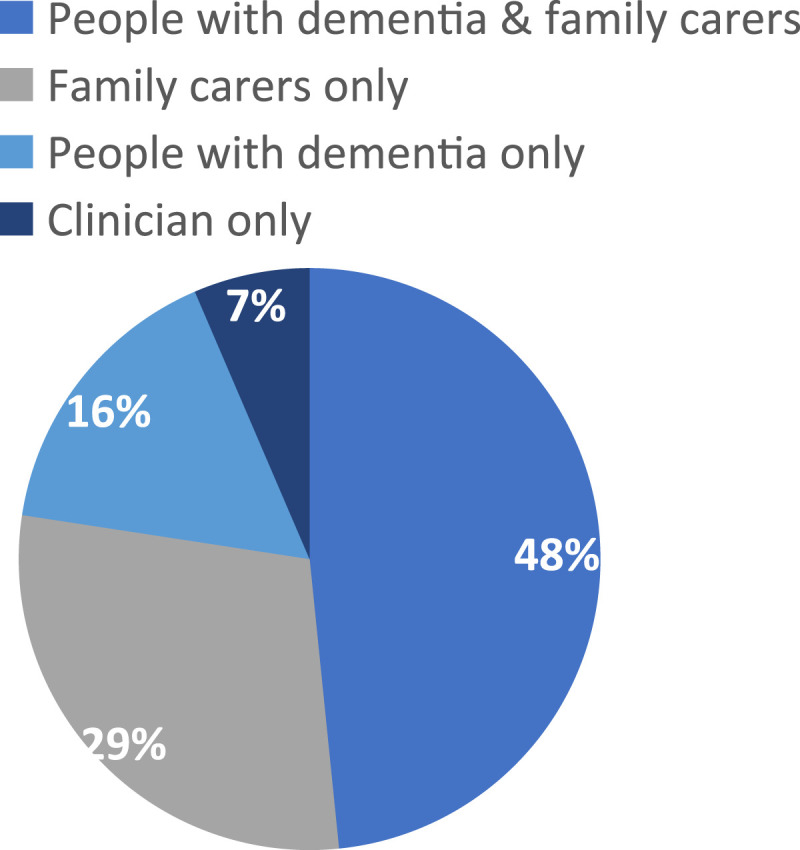
Pie chart showing who goals were set with via % of the included studies (*n* = 30).

**Figure 3.**
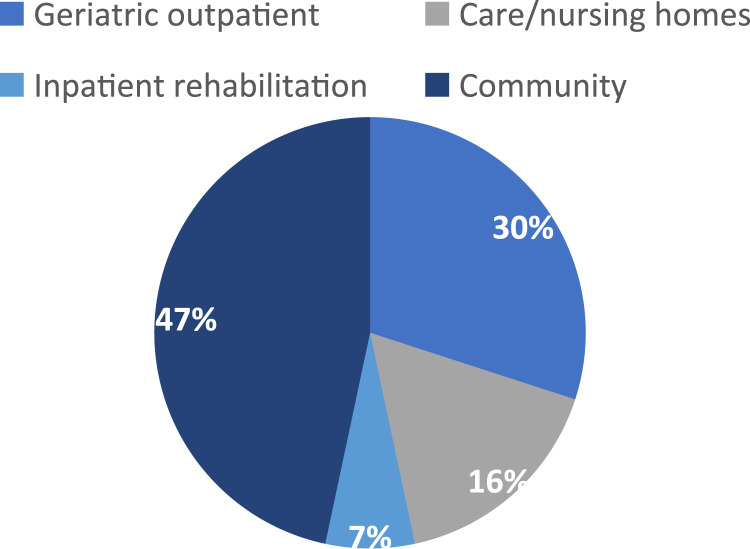
Pie chart showing study setting via % of the included studies (*n* = 30).

**Figure 4.**
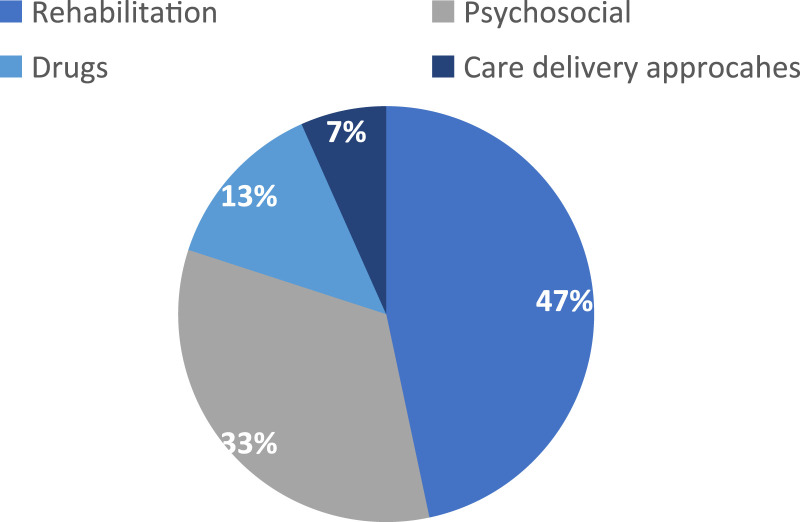
Pie chart showing the type of interventions tested via % of the included studies (*n* = 30).

Table 2 outlines the study characteristics and outcome measure characteristics. Figures 2–4 describe included studies in terms of who set the goals with facilitators, the study setting, and the types of interventions being tested.

### Goal-orientated measures used

Four named goal-setting outcome measures were identified in the included articles (Table 2); Goal Attainment Scaling (GAS) (*n* = 19; 63%), the Bangor Goal-Setting Interview (BGSI) protocol (*n* = 3; 10%), the Canadian Occupational Performance Measure (COPM) (*n* = 2, 7%), and the Individually Prioritized Problems Assessment (IPPA) (*n* = 1; 3%). Five studies used study-specific goal setting methods (17%). One study used a combination of COPM and GAS.

### Goal attainment scaling

Goal attainment scaling was used to test psychosocial interventions (*n* = 10), rehabilitation (*n* = 5) and drug studies (*n* = 4) (see Table 2). In most studies, clinicians facilitated the goal setting and scored attainment (*n* = 14), experienced dementia care staff facilitated GAS in five studies and one study used non-clinically trained facilitators (Rapaport et al., 2021). In ten studies, family carers were the primary person involved in GAS but three of these studies included the people living with dementia wherever possible. Five studies explicitly involved both the people living with dementia and family carers. The people living with dementia was the primary person setting goals in two studies; in one of them the family carer was involved if available. Two geriatricians and a nurse collaborated to set goals on behalf of care home residents in one study (Gordon et al., 1999).

Goal attainment scaling formulates individualised scoring scales when setting the goal, usually defining what the baseline level of behaviour would look like if it were to get ‘much worse’, ‘worse’, ‘better’ or ‘much better than expected’. All but one study used a 5-point scale to assess goal attainment (usually −2 to +2, although two studies used ratings of −1 to +3 (Wilz et al., 2011; Wilz et al., 2018), while the remaining study used a 3-point scale; 0 (no change) to 2 (completely achieved) (Harris et al., 2020). There was also variation in values ascribed to the scale numbers. The original GAS methodology (Kiresuk & Sherman, 1968) allocates the baseline level at ‘−1’ or ‘−2’ with ‘0’ being ‘goal achieved’. In eight studies ‘zero’ was defined as ‘goal achieved’ or ‘expected outcome’. In seven studies, zero was ascribed to the baseline status or current level of functioning, which allows for more levels of deterioration which may be more suitable for degenerative diseases like dementia where decline is more likely (Rockwood et al., 2002). Two studies did not specify the scaling used (Petyaeva et al., 2018; Watchman et al., 2021).

The GAS follow-up periods ranged from 1 week to 12 months. Five studies asked participants to rank goals in order of importance or priority and used rankings to weight scores. One study (Jennings et al., 2018) asked people living with dementia and family carers to rate how difficult they thought their goals would be to achieve on a four-point scale (not at all difficult to extremely difficult). 10/19 studies transformed GAS ratings into GAS T-scores using a standardised formula (Kiresuk & Sherman, 1968). Other studies used narrative methods to report on number and type of goals achieved, and attainment levels.

### Other goal-setting measures

The COPM was used in two RCTs (Clare et al., 2010; Regan et al., 2017) that test cognitive rehabilitation interventions. COPM provides a semi structured interview format to help users identify goals within selected areas. COPM uses a 10-point scale to measure the level of performance and satisfaction with goal attainment (1; unable to perform/not satisfied, to 10; fully able to perform/extremely satisfied). The ‘change score’ is calculated by summing the individual goal ratings of performance and satisfaction and then dividing by the number of goals set. In both studies non-clinically trained but supervised research assistants facilitated the goal setting with people living with dementia only. The follow up periods for COPM are shorter than the other measures at 4 weeks and 8 weeks.

The BGSI is used in a series of three studies (Watermeyer et al., 2016) testing cognitive rehabilitation programmes by the research group who developed BGSI (Clare et al., 2015). Like the COPM, it is a semi structured interview and uses the same 10-point scale. It has since been used outside of this research group in a small sample of three Irish patients with Alzheimer’s Disease (Kelly et al., 2019). The BGSI is facilitated by researchers in the included studies. Clare et al. (2019) trained research assistants but the training researchers received in the other two studies is unclear. Unlike GAS, both the BGSI and the COPM use standardised, rather than individually tailored scaling systems across studies. The follow-up periods varied from 11 weeks post baseline to 9 months.

Table 2 outlines 5 goal-setting measures that have not been employed in more than one study; five developed their own measure and one non-randomised study (Bemelmans et al., 2016) used the IPPA which is a measure specially developed to assessed the effectiveness of assistive technology (Wessels et al., 2002).

### Findings from studies employing goal-orientated measures in randomised control trials

Out of the eleven RCTs, six utilised GAS and five used COPM (*n* = 2), BGSI (*n* = 2) or a self-developed method (*n* = 1). Three of the RCTs using GAS (Boots et al., 2017; Chester et al., 2021; Wilz et al., 2011) were not assessed for quality or psychometric properties since they only used GAS in their intervention group or as part of the intervention.

#### Quality appraisal of randomised control trials

Table 3 outlines both the Risk of Bias and quality ratings for the goal setting measures in each RCT. Table 4 summarises the overall quality of the measurement properties within the RCTs following the modified GRADE approach.

**Table 3.** Risk of Bias ratings and measurement property quality ratings within RCTs (Cells highlighted in green indicate the psychometric property was given a good or adequate risk of bias rating and are of sufficient quality).

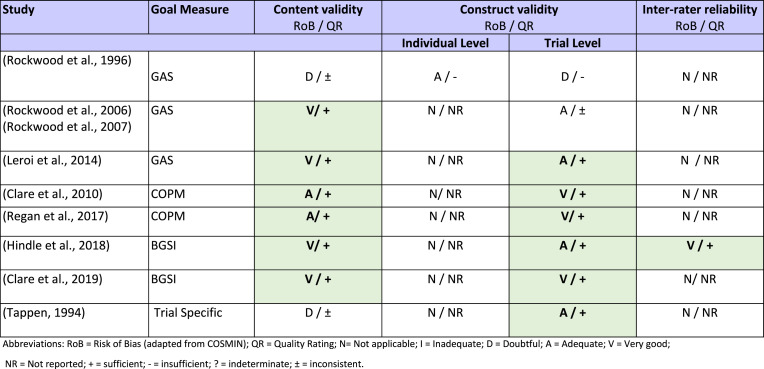

**Table 4.** Overall rating and quality of evidence of goal outcome measures in RCTs

	GAS		COPM		BGSI	
	Overall rating	Quality of evidence	Overall rating	Quality of evidence	Overall rating	Quality of evidence
	+/−/?	High, moderate, low, very low	+/−/?	High, moderate, low, very low	+/−/?	High, moderate, low, very low
Content validity	+	Moderate	+	Moderate	+	Moderate
Construct validity (individual level)	?	Low	?	Low	?	Low
Construct validity (trial level)	+	Moderate	+	Moderate	+	Moderate
Reliability	?	Moderate	?	Low	+	Moderate

Abbreviations: + = sufficient; − = insufficient; ? = indeterminate; ± = inconsistent.

#### Content validity

All eight RCTs evaluated content validity and across the studies GAS, COPM and BGSI was rated as sufficient, with a moderate level of available evidence due to the small number of RCTs in total (Table 4). Table 3 shows that sufficient evidence for content validity was reported for two studies using GAS (Leroi et al., 2014; Rockwood et al., 2006) and two studies using the BGSI (Clare et al., 2019; Hindle et al., 2018). These studies described the methodology in detail and synthesised the mixed methods. The goal setting and scoring were reviewed by independent experts and both the people living with dementia and family carers were involved in the setting and scoring of goals. There was also a focus on ensuring that goals were based on SMART criteria. The two RCTs using COPM (Clare et al., 2010; Regan et al., 2017) had adequate and sufficient evidence for content validity. Two RCTs indicated doubtful content validity and were of inconsistent quality (Rockwood et al., 1997). The level of content analysis performed on set goals varied between the RCTs, ranging from detailed analysis that outlined the goal domains, areas and examples (Clare et al., 2019; Hindle et al., 2018; Rockwood et al., 2006), to brief summaries of overall goal domains (Clare et al., 2010; Leroi et al., 2014; Regan et al., 2017; Rockwood et al., 1996), to no analysis of the content being performed (Tappen, 1994).

#### Construct validity

Construct validity was assessed at individual and trial levels. Only one study was assessed on an individual level but was rated to have insufficient quality. Rockwood et al. (1996) found that GAS scores were found to correlate moderately with ADAS-Cog (a measure of cognitive ability, r = .52) and GDS (Global Deterioration Scale, r = .63) but not with MMSE (Mini mental state examination, r = .004) (Rockwood et al., 1996). Thus, for GAS, COPM and BGSI, there was insufficient evidence to draw a conclusion on an individual level.

On a trial level, there was overall sufficient evidence of moderate-quality for the construct validity of COPM, GAS, BGSI and an unspecified measure, see Table 4. It is expected that the mean GAS scores of two randomised groups receiving effective or non-effective interventions will differ in favour of the group receiving the effective intervention. All but one study found this to be the case. Rockwood et al. (1996) found no significant difference between groups (*p* = .54), but the study was exploratory with a small sample size and GAS was still concluded to be the most responsive measure with the largest effect size (.61) and relative efficacy (.47). Four studies (Hindle et al., 2018; Leroi et al., 2014; Rockwood et al., 2006; Tappen, 1994) found evidence of trial level construct validity but had small sample sizes. Three remaining studies (Clare et al., 2010, 2019; Regan et al., 2017) using the COPM or BGSI showed good methodological quality and strong evidence of good construct validity.

#### Reliability

The only type of reliability that was assessed was inter-rater reliability in one study. A pilot RCT testing a cognitive rehabilitation intervention (Hindle et al., 2018; Watermeyer et al., 2016) used an independent researcher to code a subsample of the qualitative data set from the BGSI goal setting discussions and create their own goal rating. They found excellent inter-rater reliability (Krippendorff’s alpha = .95) and agreement was 95.7%.

#### Feasibility and interpretability

One of the earliest studies did not report on feasibility (Tappen, 1994) but all proceeding RCTs reported that GAS, COPM and BGSI were feasible goal-setting measures for both facilitators and users, with all included participants able to set at least one goal. Across the RCTs, the mean number of goals was approximately three per user. Researchers using GAS met with the participants in clinical settings and those using COPM and BGSI met participants in their own homes (all in person). Only one RCT mentioned how long the goal setting process took (2 hours over 3 visits) and suggests this extra time allowed more efficient follow up interviews (Rockwood et al., 1996). No studies mention any cost associated with these outcome measures. There was a very low rate of missing data for the goal measures suggesting good interpretability. The highest percentage of missing data was 9.4% (or 12 participants in placebo group) who did not complete GAS scores at 6 and 8 months (Rockwood et al., 2006).

## Discussion

We identified four main goal setting measures being used as outcomes in this population: GAS, BGSI, COPM and IPPA. GAS, BGSI and COPM were used in RCTs, and using an adapted methodology based on COSMIN, we found moderate quality evidence for sufficient feasibility and validity, but reliability needs to be further assessed.

A central part of all three measures is the identification of goals. The BGSI and COPM focus on an initial interview in which facilitators help users identify and set goals. The COPM is based on the Canadian Model of Occupational Performance and Engagement (COPM-E) in which the client centred approach is central (McColl et al., 2005). COPM was developed for occupational therapy clinics, while the BGSI has been developed primarily as a research tool based on the concept of motivational interviewing and the social cognitive theory of behaviour change (Clare et al., 2012). The GAS studies used different approaches to identify goals; one used the COPM method (Ciro et al., 2014), others used similar interview techniques to the COPM and BGSI (Boots et al., 2016, 2017; Rockwood et al., 2006), and others used a goal inventory for clients to select goals from predefined goal areas (Jennings et al., 2018; Leroi et al., 2014; Rapaport et al., 2021). GAS differs from the other goal setting measures due to the formulation of individualised scoring scales when setting the goal. People living with dementia and family carers define in their own words what the baseline level behaviour or situation would look like it was to improve or get worse to form the 5-point scale (much worse to much better). This increases the complexity of goal setting but has the benefit that a highly personalised outcome measure is produced.

We found evidence of very good or adequate content and construct validity for GAS, BGSI and COPM in RCTs. An important consideration in evaluating content validity is assessing whether the target population was involved in setting the goals and whether the goals were reviewed by one or more independent experts (Gaasterland et al., 2016, 2019). The fact that people living with dementia are encouraged to share their preferences is important for the clinical relevance of these measures. Where people living with dementia lack capacity, studies often asked the family carer to help set the goals. Although this allows for family carer bias, the goals are still highly relevant, as they are usually the person most able to understand the people living with dementia’s preferences if they no longer have capacity to express these (de Vugt et al., 2003). We agree with the conclusion of Bouwens et al. (2008) that family carers should not only help set the goals for the people living with dementia but should also set goals relevant to themselves.

All identified goal setting measures in this study have been facilitated by either clinically trained individuals or trained and supervised research assistants. It is vital that the goals selected are relevant for the intervention, and that this should be evaluated by an expert in the intervention content (Gaasterland et al., 2019). Although it is agreed across studies that training in facilitating the measures is important, there is a lack of detail in what the training entailed. Rockwood et al. (2006) outlined that they provided 4 hours of training for health professionals and Rapaport et al. (2021) reported that the study team received 2 days of training by GAS experts. The GREAT RCT detailed an initial two-day training course, annual refresher training days and monthly supervision for optimising the goal setting process (Clare et al., 2019). Future studies should outline what training was provided, the background experience of the facilitators and levels of supervision or goal review process (if any) provided to ensure studies set suitable goals which are central to the validity of the measure.

Another important aspect of content validity was to assess whether the goals set were SMART and if any content analysis was carried out on the set goals. Determining whether putative goals are realistic may be especially challenging for people living with dementia, and access to resources must be carefully considered. Ensuring goals are SMART is explicitly written into the guidance for COPM and BGSI. The GAS methodology has evolved to include the importance of setting well defined SMART goals at baseline (Rockwood et al., 1996; Tappen, 1994). The level of content analysis performed on set goals varied between studies, but it is recommended that goal content analysis is completed where goal domains, areas and descriptors are clearly outlined (Gaasterland et al., 2019).

The construct definition and the score meaning is crucial to determine if change is effectively measured on an individual level. It is therefore easier to assess construct validity on a trial level where two groups can be compared and where it is expected that the change scores of the two randomised groups will differ in favour of the one receiving the effective intervention (Gaasterland et al., 2019). All included RCTs formulated specific hypotheses and provided an adequate description of the intervention for construct validity to be assessed.

Reporting on reliability of the goal setting measures was limited in the RCTs but more common in some of the non-RCT studies. Although reliability contributes to the accuracy of findings, it cannot be a substitute for validity (Zumbo et al., 2014). Only one RCT study demonstrated excellent inter-rater reliability of the BGSI (Hindle et al., 2018; Watermeyer et al., 2016) by having a second independent researcher complete the goal content analysis of baseline goals. One aspect of evaluating reliability is determining whether the time intervals were appropriate or not. Goal outcome measures have been used up to twelve months follow up in previous nonrandomised studies (Boots et al., 2017; Jennings et al., 2018; Judge et al., 2011; Rockwood et al., 2002) for PWLD but it is not known what would be deemed an inappropriate time interval for these measures for people living with dementia. GAS has been used within RCTs with this population up to 6 months (Chester et al., 2021; Ciro et al., 2014) and BGSI has been used up to 9 months, but the primary outcome was still set at 3 months (Clare et al., 2019). Future work could explore using these goal measures over different time periods to determine the optimal time for follow ups which is likely to be dependent on dementia severity. This work would help further establish the reliability and feasibility of these outcomes and determine when goals are no longer relevant or of insignificant importance to the users.

The identified outcome measures have been used for interventions aimed at people living with dementia and/or family carers across a wide variety of dementia diagnosis types, mainly of mild-moderate severity although some studies have included people of all severities (often in care home settings; (Petyaeva et al., 2018)). In studies that set goals with people living with dementia and family carers it is sometimes unclear what the level of people living with dementia engagement was. When adapting these goal setting systems, it should be considered if there are ways to maximise the contribution of people living with dementia. A study in Japan used the Aid for Decision-making in Occupation Choice (ADOC) measure (Tomori et al., 2012) to ask people living with dementia to select 20 activities from 95 illustrations of daily activities which was then reduced to the 5 most important. The use of illustrations or other adapted methods in the goal selection process is unexplored. All the goals set and rated with participants in this review have been done in person, face to face within clinics, care homes or the participants’ own homes. At the time of writing, the COVID-19 pandemic resulted in a shift in how people interact and so it would be timely to explore the psychometric properties of goal setting measures completed via remote methods too.

Goal setting outcomes enables research to use personalised outcomes to assess the efficacy of interventions, but they can also serve to tailor interventions and be part of the intervention. Goal setting is a crucial aspect in rehabilitation settings for example (Turner-Stokes et al., 2018). Rapaport et al. (2021) used GAS as both a primary outcome measure but also to directly inform the psychosocial intervention (NIDUS-Family) for PWLD and their family carers. There are several benefits to setting goals, including helping family carers and people living with dementia communicate priorities and needs (Jennings et al., 2018), keeping motivation high leading to better performance and prolonged effort as well as improving people’s sense of self-efficacy (Locke & Latham, 2002). Further work in validating and establishing the best methodology of goal setting outcome measures may also have benefits to evaluate person-centred care within clinical and social care settings. Future reviews may also consider including studies that recruited paid/professional home care workers since we know how crucial a dedicated and adequate supported home-care workforce is to quality care (Carter, 2016).

Although we include studies from four continents, participants were all from high-income countries and were generally highly educated which limits the generalisability of the findings. Future studies should look to implement goal outcome measures with people living with dementia and family carers with lower socioeconomic status and educational attainments. One of the major advantages of goal setting measures is that they can account for differing cultural norms and languages in a way other standardised questionnaires cannot.

While we followed methods developed in previous studies, there is still a lack of agreement or a standardised way to assess the psychometric properties of goal outcome measures. There are limitations to trying to adapt the COSMIN guidance to apply to these unique types of outcome measures. We were only able to evaluate limited psychometric properties in a limited number of RCTs. With each measurement only being used by 1-3 RCTs we are unable to assess the influence of any of the psychometric properties. Further RCTs within dementia research utilising goal setting outcome measures will be crucial for further evaluation.

## Conclusion

This study shows there is adequate evidence of content and construct validity and feasibility for GAS, BGSI, COPM being used as goal-setting measures for people living with dementia and family carers in RCTs. There is good evidence of inter-rater reliability for BGSI in one RCT, but reliability is not tested in other RCTs. We are not able to conclude on the use of one measure over another but suggest that GAS, BGSI and COPM have different strengths. The BGSI and COPM provide good guidance on an effective approach to goal identification interviews while GAS provides a detailed and personalised scaling system that is designed to be particularly sensitive to change. The flexibility and adaptability of goal setting measures can be beneficial for dementia researchers, as shown by the studies which developed their own goal attainment systems.

A key feature of goal setting outcome measures is that it enables people living with dementia and family carers to select goals within variable life domains that can reflect the high multidimensionality of dementia and that can be selected to fit the intervention, project or setting. There is no recommended guide of how to use GAS with people living with dementia so training and practice in how to set goals with this population and their carers is important. Further development and recommendations for facilitator training could be a beneficial way to ensure individualised person-centred outcomes are more widely used in RCTs while also allowing further evaluation of the psychometric properties of these measures.

## Supplemental Material

Supplemental Material - Setting individualised goals for people living with dementia and their family carers: A systematic review of goal-setting outcome measures and their psychometric properties Supplemental Material for Setting individualised goals for people living with dementia and their family carers: A systematic review of goal-setting outcome measures and their psychometric properties by Jessica Budgett, Andrew Sommerlad, Nuriye Kupeli, Sedigheh Zabihi, Anna Olsen and Claudia Cooper in Dementia.
