# Supplementary material for: Setting individualised goals for people living with dementia and their family carers: A systematic review of goal-setting outcome measures and their psychometric properties
Source: Dementia (London). 2023 Dec 17;23(2):312–40. doi: 10.1177/14713012231222309 (PMC10807246; doi:10.1177/14713012231222309)
Supplement: Supplemental Material - Setting individualised goals for people living with dementia and their family carers: A systematic review of goal-setting outcome measures and their psychometric properties [file sj-pdf-1-dem-10.1177_14713012231222309.pdf]

Appendix 1: Search strategy keywords

Block 1: Dementia

("dementia"[MeSH Terms] OR "dementia"[All Fields] OR "dementias"[All Fields] OR "dementia s"[All Fields] OR ("alzheime s"[All Fields] OR "alzheimer disease"[MeSH Terms] OR ("alzheimer"[All Fields] AND "disease"[All Fields]) OR "alzheimer disease"[All Fields] OR "alzheimer"[All Fields] OR "alzheimers"[All Fields] OR "alzheimer s"[All Fields] OR "alzheimers s"[All Fields]))

Block 2: Goals

AND ("goals"[MeSH Terms] OR "goals"[All Fields] OR "goal"[All Fields])

Block 3: Outcome measures

AND ("measurability"[All Fields] OR "measurable"[All Fields] OR "measurably"[All Fields] OR "measure s"[All Fields] OR "measureable"[All Fields] OR "measured"[All Fields] OR "measurement"[All Fields] OR "measurement s"[All Fields] OR "measurements"[All Fields] OR "measurer"[All Fields] OR "measurers"[All Fields] OR "measuring"[All Fields] OR "measurings"[All Fields] OR "measurment"[All Fields] OR "measurments"[All Fields] OR "weights and measures"[MeSH Terms] OR ("weights"[All Fields] AND "measures"[All Fields]) OR "weights and measures"[All Fields] OR "measure"[All Fields] OR "measures"[All Fields] OR ("questionnair"[All Fields] OR "questionnaire s"[All Fields] OR "surveys and questionnaires"[MeSH Terms] OR ("surveys"[All Fields] AND "questionnaires"[All Fields]) OR "surveys and questionnaires"[All Fields] OR "questionnaire"[All Fields] OR "questionnaires"[All Fields]) OR "tool"[All Fields] OR ("scale s"[All Fields] OR "scaled"[All Fields] OR "scaling"[All Fields] OR "scalings"[All Fields] OR "weights and measures"[MeSH Terms] OR ("weights"[All Fields] AND "measures"[All Fields]) OR "weights and measures"[All Fields] OR "scale"[All Fields] OR "scales"[All Fields]) OR ("instrument"[All Fields] OR "instrument s"[All Fields] OR "instrumentation"[MeSH Subheading] OR "instrumentation"[All Fields] OR "instruments"[All Fields] OR "instrumented"[All Fields] OR "instrumenting"[All Fields]) OR ("assess"[All Fields] OR "assessed"[All Fields] OR "assessement"[All Fields] OR "assesses"[All Fields] OR "assessing"[All Fields] OR "assessment"[All Fields] OR "assessment s"[All Fields] OR "assessments"[All Fields]) OR ("inventoried"[All Fields] OR "inventory s"[All Fields] OR "inventorying"[All Fields] OR "personality inventory"[MeSH Terms] OR ("personality"[All Fields] AND "inventory"[All Fields]) OR "personality inventory"[All Fields] OR "inventories"[All Fields] OR "equipment and supplies"[MeSH Terms] OR ("equipment"[All Fields] AND "supplies"[All Fields]) OR "equipment and supplies"[All Fields] OR "inventory"[All Fields]))

Appendix 2: Adapted COSMIN Risk of Bias (Mokkink et al., 2018) boxes to determine overall quality studies using goal-setting outcome measures.

CONTENT VALIDITY	Very Good	Adequate	Doubtful	Inadequate	Not applicable
1.Were the goals set reviewed or evaluated by one or more independent experts?<br>Experts can be considered broadly and may include clinicians, patients, or family members**.**<br>***(Gaasterland et al., 2019)***	The goals set were reviewed or evaluated by one or more independent experts and the method in which this was done is clearly stated.	Assumable that the goals set were reviewed or evaluated by one or more independent experts.	Not clear if goals set were reviewed or evaluated by one or more independent expert or there were no reviewers of the goals set.	*	
2.Were the goal achievement levels/scale evaluated by independent clinical experts.<br>***(Gaasterland et al., 2019)***	The goal achievement levels/scale was evaluated by independent clinical experts and the method in which this was done is clearly stated.	Assumable that the goal achievement levels/scale was evaluated by independent clinical experts	Not clear if the goal achievement levels/scale was evaluated by independent clinical experts or there were no independent evaluators.	*	
3.Was the target population (PLWD or FCs) involved in goal selection/setting?<br>***(Gaasterland et al., 2016)***	The target population was involved in the goal selection/ setting	Assumable that the target population was involved OR they were not involved but a suitable explanation to why not is provided	Not clear if the target population was involved in the goal selection/ setting or they were not involved, and no explanation provided	**	
4.Were goals formulated according to the criteria 'Specific, Measurable, Attainable, Realistic, and Time-bound' (SMART)<br>***Gaasterland et al. (2016)***	The SMART criteria was checked by second reviewer.	Some elements of SMART criteria or similar were applied to goal setting but it may not mention whether this was checked by an independent evaluator.	Not clear if any SMART criteria was applied to the goals.	*	

5.Were the goal areas summarised or coded into goal areas or goal domains?<br>Evidence of content analysis of identified goal areas.<br>**(Shankar et al., 2020)**<br>**(Bouwens et al., 2008)**	Clear evidence of some form of content analysis of identified goal areas – the results of this is provided.	Some evidence of some form of content analysis of identified goal areas – but results may not be presented in the paper.	Not clear if any content analysis of goal areas was done.	*	
6.Were skilled facilitators used or were facilitators/raters suitably trained?<br>**(Adapted from COMSIN (Mokkink et al., 2018))**	Evidence of facilitators receiving suitable amount of training (mentions training sessions or role play practice).<br><br>OR clear that facilitators were already sufficiently skilled.	Facilitators had limited training or experience in using goal outcome measure.<br><br>Or facilitators had limited experience in the dementia field	Not clear if facilitators were trained or not trained.	Evidence that facilitators were not appropriately skilled	

Abbreviations: PLWD, People Living with Dementia; FCs, Family carers; SMART (specific, measurable, attainable, realistic, and time-bound)

* Item is considered ideal or recommended to evaluate content validity but it is not an essential or well described process of the GAS method and therefore we do not require the study to be listed as inadequate if it does not do this but listed as doubtful instead.

** Having the target population involved in the setting or selection of goals is ideal but is not an essential part of GAS and in some studies valid explanations of why they were not included are provided.

**CONSTRUCT VALIDITY (Trial Level, hypothesis testing)**	**Very Good**	**Adequate**	**Doubtful**	**Inadequate**	**Not applicable**

1.Were specific hypotheses formulated? ***(Gaasterland et al., 2019)***	Adequate description of the hypotheses		Poor description of the hypotheses	NO description of the hypotheses	
2.Was an adequate description provided of the intervention given? **(COSMIN Box 10.d (Mokkink et al., 2018))**	Adequate description of the intervention		Poor description of the intervention	NO description of the intervention	
3.Was the statistical method appropriate for the hypotheses to be tested? ***(COSMIN, Box 10d*** **(Mokkink et al., 2018)*)***	Statistical method was appropriate	Assumable that statistical method were appropriate	Statistical method applied NOT optimal	Statistical method applied NOT appropriate	
4.Were there any other important flaws in the design or statistical methods of the study? (**COSMIN, Box 10.d (Mokkink et al., 2018))**	No other important methodological flaws		Other minor methodological flaws	Other important methodological flaws	
**CONSTRUCT VALIDITY (Individual level / Comparison with secondary measure)**	**Very Good**	**Adequate**	**Doubtful**	**Inadequate**	**Not applicable**

1.Is it clear what the comparator instrument(s) measure? ***(COSMIN, Box 10b (Mokkink et al., 2018))***	Constructs measured by the comparator instrument(s) is clear			Constructs measured by the comparator instrument(s) is not clear	
2.Were the measurement [illegible]	Sufficient measurement [illegible]	Sufficient measurement [illegible]	Some information on measurement [illegible]	NO information on the measurement [illegible]	

**RELIABILITY (COSMIN, box 6 (Mokkink et al., 2018))**	**Very Good**	**Adequate**	**Doubtful**	**Inadequate**	**Not applicable**
1.Were the test conditions similar for the measurements? E.g type of administration, environment, instructions	Test conditions were similar (evidence provided)	Assumable that test conditions were similar	Unclear if test conditions were similar	Test conditions were NOT similar	
2.Was the time interval appropriate?	Time interval appropriate		Doubtful whether time interval was appropriate or time interval was not stated	Time interval NOT appropriate	
3.For continuous scores: Was an intraclass correlation coefficient (ICC) calculated?	ICC calculated and model or formula of the ICC is described	ICC calculated but model or formula of the ICC not described or not optimal. Pearson or Spearman	Pearson or Spearman correlation coefficient calculated WITHOUT evidence	No ICC or Pearson or Spearman correlations calculated	Not applicable

		correlation coefficient calculated with evidence provided that no systematic change has occurred	provided that no systematic change has occurred or WITH evidence that systematic change has occurred		
4.For dichotomous/nominal/ordinal scores: Was kappa calculated?	Kappa calculated			No kappa calculated	Not applicable
5.For ordinal scores: Was a weighted kappa calculated?	Weighted Kappa calculated		Unweighted Kappa calculated or not described		Not applicable
6.For ordinal scores: Was the weighting scheme described? e.g. linear, quadratic	Weighting scheme described	Weighting scheme NOT described			Not applicable
7.Were there any other important flaws in the design or statistical methods of the study?	No other important methodological flaws		Other minor methodological flaws	Other important methodological flaws	

Bouwens, S. F., van Heugten, C. M., & Verhey, F. R. (2008). Review of goal attainment scaling as a useful outcome measure in psychogeriatric patients with cognitive disorders. *Dement Geriatr Cogn Disord*, *26*(6), 528-540. https://doi.org/10.1159/000178757

Gaasterland, C. M., Jansen-van der Weide, M. C., Weinreich, S. S., & van der Lee, J. H. (2016). A systematic review to investigate the measurement properties of goal attainment scaling, towards use in drug trials. *BMC Med Res Methodol*, *16*, 99. https://doi.org/10.1186/s12874-016-0205-4

Gaasterland, C. M. W., van der Weide, M. C. J., Roes, K. C. B., & van der Lee, J. H. (2019). Goal attainment scaling as an outcome measure in rare disease trials: a conceptual proposal for validation. *BMC Med Res Methodol*, *19*(1), 227. https://doi.org/10.1186/s12874-019-0866-x

Mokkink, L. B., de Vet, H. C. W., Prinsen, C. A. C., Patrick, D. L., Alonso, J., Bouter, L. M., & Terwee, C. B. (2018). COSMIN Risk of Bias checklist for systematic reviews of Patient-Reported Outcome Measures. *Qual Life Res*, *27*(5), 1171-1179. https://doi.org/10.1007/s11136-017-1765-4

Shankar, S., Marshall, S. K., & Zumbo, B. D. (2020). A systematic review of validation practices for the goal attainment scaling measure. *Journal of Psychoeducational Assessment*, *38*(2), 236-255. https://doi.org/10.1177/0734282919840948

References
